# Coccidioidomycosis Causing Hydrocephalus

**DOI:** 10.7759/cureus.21889

**Published:** 2022-02-03

**Authors:** Mani Maheshwari, Shashikala Ameneni, Hemanthkumar Athiraman

**Affiliations:** 1 Hospital Medicine, Banner Health, Mesa, USA; 2 Infectious Disease, Banner Health, Mesa, USA

**Keywords:** immunocompromised patient, evd, desert rheumatism, san joaquin valley fever, coccidioidomycosis

## Abstract

Coccidoidomycosis is caused by *Coccidoides imitis* and *C. posadasii* infection. Coccidioidomycosis is also known as San Joaquin Valley fever or desert rheumatism. It is only seen in the Southwest United States (Arizona, New Mexico, California, Texas, Nevada, and Utah), and Central and South America. This infection is acquired by the inhalation of fungal spores in the air. The most severe extrapulmonary coccidioidomycosis is coccidioidomycosis meningitis, in which patients present with headaches, photophobia, altered mental status, and hearing difficulties. This is a case report of a person with disseminated coccidioidomycosis meningitis complicated by hydrocephalus, presenting as a headache.

## Introduction

*Coccidioides immitis* and *C. posadasii* are coccidioidomycosis-causing dimorphic fungi found in the Southwestern United States and in Central and South America [1]. There is a lack of awareness regarding coccidioidomycosis in these endemic areas, and it is often missed, although about 30% of community-acquired pneumonia cases are due to coccidioidomycosis [2,3]. Coccidioidomycosis is at times managed supportively. The decision to treat rests on many factors, including disease severity, imaging findings, anticomplementary titers, immunosuppression or immunocompromised state, comorbidities [1]. The risk factors for disseminated coccidioidomycosis are immunosuppression, African or Filipino descent, and pregnancy [4]. There are about 150,000 coccidioidomycosis infections every year, with about 150 of these infections going on to disseminate into coccidioidomycosis meningitis [5].

This case report describes the most lethal type of disseminated coccidioidomycosis, meningitis, in a patient with latent tuberculosis and hepatitis C infection, both of which had not been treated. It is diagnosed using lumbar puncture/spinal tap, with cerebrospinal fluid analysis showing elevated WBC with lymphocytic pleocytosis, high protein, low glucose, and positive coccidioidomycosis serology with complementary fixation titers [6]. Titers of less than 1:16 indicate past infection or self-limited disease, and more than 1:16 indicates disseminated infection [7].

## Case presentation

**Figure 1:**
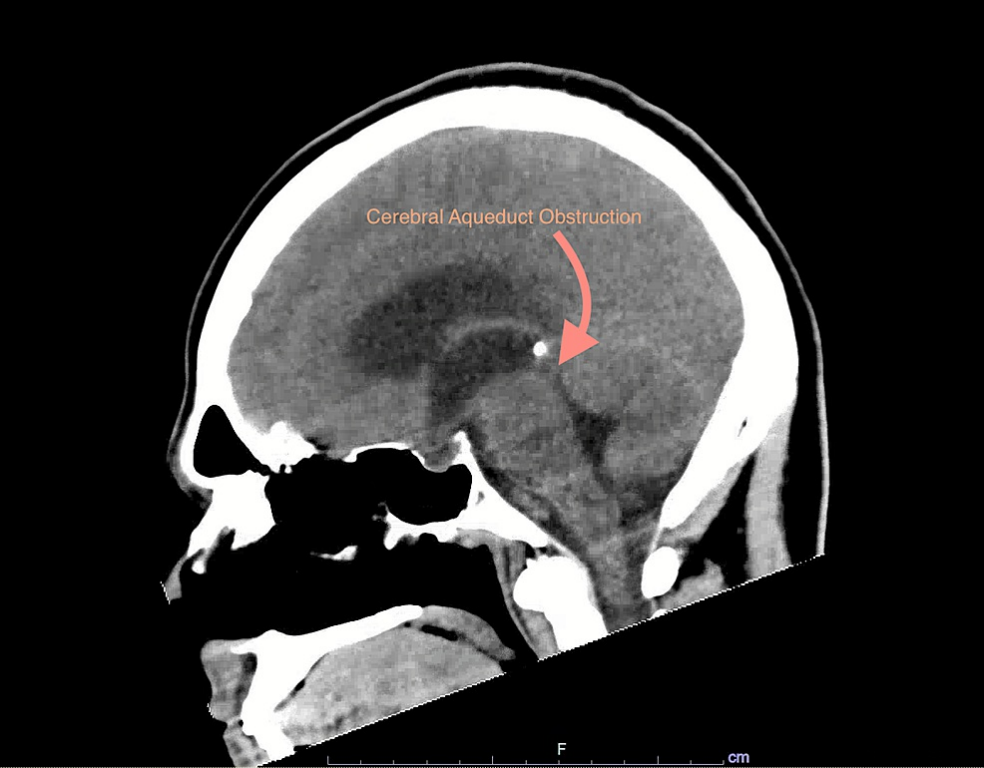
CT head/brain/cervical spine shows hydrocephalus with obstruction at cerebral aqueduct

A 39-year-old incarcerated male with a past medical history of hepatitis C, asthma, hypercholesterolemia, and posttraumatic stress disorder presents with three months of headache and pre-syncope without prior workup. He states that the headache worsens when he eats, has intermittent photophobia, has blurry vision, and has difficulty focusing for 30 seconds. The patient's vital signs in the ER were as follows: blood pressure of 112/91 mmHg, heart rate of 72 bpm, respiratory rate of 18 breaths/min, pulse oximetry of 100% on room air, and an oral temperature of 36.9 °C. Laboratory assessment is shown in Table 1. A chest X-ray shows no abnormalities. A CT scan of the head/brain/cervical spine shows hydrocephalus with obstruction at the cerebral aqueduct and a right upper lobe pulmonary nodule (Figure 1). The Glasgow Coma Scale score is 15.

**Table 1:** Laboratory assessment on admission

Lab	Patient’s result	Normal result/range
White blood cell count (K/µL)	8.9	4.0–11.0
Hemoglobin (g/dL)	13.5	13.5–17.0
Hematocrit (%)	41.5	40.0–53.0
Platelet (K/µL)	408	130–450
Glucose (mg/dL)	105	70–106
Blood urea nitrogen (mg/dL)	7	8–25
Creatinine (mg/dL)	0.54	0.60–1.50
Sodium (mmol/L)	137	134–147
Aspartate transaminase (U/L)	14	12–47
Alanine transaminase (U/L)	22	5–60

The patient was admitted to the intensive care unit and empirically treated with IV acyclovir 700 mg Q8h, IV amphotericin B 350 mg Q24h, IV daptomycin 425 mg Q24h, IV fluconazole 800 mg Q24h, and IV meropenem 2 g Q8h. The results of the lumbar puncture/CSF analysis are shown in Table 2.

**Table 2:** Lumbar puncture results (CSF analysis)

Cerebrospinal fluid lab	Patient’s result	Normal result/range
Spinal fluid color and character	Colorless and slightly hazy	Colorless and clear
White blood cell count	553/mm^3^	0-5/mm^3^
Red blood cell count	120/mm^3^	0-10/mm^3^
Neutrophils (%)	37	0–6
Lymphocytes (%)	16	40–80
Monocytes (%)	28	15–45
Glucose (mg/dL)	14	40–70
Protein (mg/dL)	583.0	15.0–40.0

The patient declined neurologically, becoming more lethargic, disoriented, and unable to awaken. Interventional radiology was emergently consulted, and an external ventricular drain (EVD) was placed. The initial transduced ICP was 25 mmHg, and the drain was kept open at 10 mmHg. CSF was draining at 165 mls/day. The microbiology data results are shown in Table 3; positive for Coccidioides in the CSF, high West Nile Virus IgG in the CSF, and QuantiFERON-TB panel showing results consistent with latent tuberculosis.

**Table 3:** Microbiology data

Microbiology lab	Patient’s result	Normal result/range
Coccidioides EIA Ab, TP (typically IgM)	Positive	Negative
Coccidioides EIA Ab, CF (typically IgG)	Positive	Negative
Coccidioides IMDF Ab, IgM (TP)	Positive	Negative
Coccidioides IMDF Ab, IgG (CF)	Positive	Negative
Coccidioides Comp Fix	1:64	Negative
Cocci Comp Fix, CSF	1:32	Negative
HCV RNA, PCR, Quant (IU/mL)	4,740,000	<15/not detected
HCV RNA, PCR, Quant (LogIU/mL)	6.68	<1.18/not detected
HSV 1 DNA, real-time PCR	Not detected	Not detected
HSV 2 DNA, real-time PCR	Not detected	Not detected
HIV 1/2 Ag/Ab screen (4th Gen)	Non-reactive	Non-reactive
West Nile Virus, IgG, CSF	3.87	Negative
West Nile Virus, IgM, CSF	<0.90/not detected	Not detected

**Figure 2:**
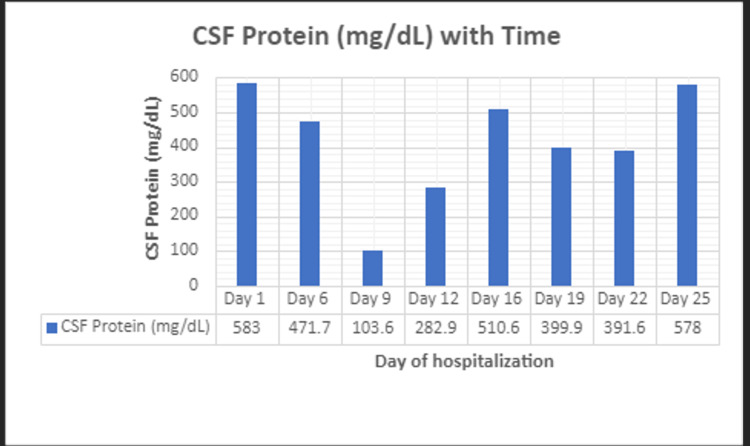
Cerebrospinal fluid protein (mg/dL) levels with time

The treatment regimen is tailored to the coccidioidomycosis meningitis/disseminated coccidioidomycosis infection, and the dose is increased to fluconazole 1,200 mg IV daily. The patient did not tolerate EVD weaning/clamp with increasing CSF protein. The CSF protein values are shown in Figure 2. The gradual increase of CSF protein levels delayed ventriculoperitoneal (VP) shunt placement because, as per neurosurgery, a VP shunt can only be placed once CSF protein levels are less than 150.

## Discussion

Coccidioidomycosis meningitis, if not treated, is universally fatal; 91% of patients will die within one year, and all patients will die within two years [8]. These numbers improve if patients are treated with azoles or Ambisome [9]. The guidelines suggest treating coccidioidomycosis meningitis with high-dose fluconazole, 800 to 1200 mg daily. No trial has compared intrathecal Ambisome and high-dose fluconazole. Although there is a delay in the normalization of CSF studies after treatment with fluconazole, symptoms generally resolve within 4-8 months. The response to treatment is assessed by the improvement in clinical symptoms and decreased coccidioidomycosis complementary fixation titers [10].

The most common complications of cocci meningitis are hydrocephalus, CNS vasculitis, infarction, vasospasm, and hemorrhage. A VP shunt is necessary for patients with hydrocephalus [10]. Furthermore, a study by Dewsnup et al. states that disease is only suppressed in coccidioidomycosis meningitis patients who achieved remission during azole treatment [11].

For the patient in this case report, after 19 days of IV fluconazole, he complained of extensive pruritis and skin sloughing. The treatment was changed to IV voriconazole, 700 mg Q12H, and the pruritis worsened. The treatment was changed to oral itraconazole 200 mg every eight hours and intrathecal amphotericin 0.1 mg Q48h. EVD was maintained open at 5 mmHg, and CSF output increased to 218 mLs/day. The gradual increase in CSF protein levels delayed VP shunt placement.

After 19 days of intrathecal amphotericin only, IV amphotericin 350 mg Q24hrs was added to the regimen since CSF white blood cell count and protein levels increased. The patient continued to have headaches.

The patient was then re-challenged with voriconazole 450 mg PO Q12h, and he tolerated it; at this point, itraconazole was discontinued. Serum and CSF coccidioidomycosis titers were checked weekly and trended down. Serum titers decreased from 1:64 to 1:16 over four weeks. CSF titers decreased from 1:64 to 1:1 over four weeks. Similarly, the CSF white blood cell count also started to trend down, and, subsequently, intrathecal Ambisome was discontinued. The EVD was clamped, and the repeat CT head was stable (no dilation of ventricles), allowing for the safe removal of the EVD.

The patient developed hallucinations, confusion, and acute transaminitis two days later due to voriconazole. Until the patient’s mental status and liver function tests improved, voriconazole was held for the next seven days. Afterward, the patient was transitioned to oral voriconazole 300 mg Q12h. Subsequently, he was cleared for discharge on IV Ambisome 350 mg Q12h for four more weeks and voriconazole 300 mg Q12h for life. More than 90 days after admission, he remains in the hospital on the same regimen.

## Conclusions

It is vital to detect coccidioidomycosis infection in patients who have recently traveled to or live in endemic areas such as Arizona, New Mexico, California, Texas, Nevada, Utah, and parts of Central and South America. Delayed diagnosis leads to unnecessary healthcare utilization and costs, improved antibiotic stewardship, and prompt treatment of patients. If left untreated, coccidioidomycosis may be fatal.
